# Postactivation Performance Enhancement (PAPE) Increases Vertical Jump in Elite Female Volleyball Players

**DOI:** 10.3390/ijerph19010462

**Published:** 2022-01-01

**Authors:** Lamberto Villalon-Gasch, Alfonso Penichet-Tomas, Sergio Sebastia-Amat, Basilio Pueo, Jose M. Jimenez-Olmedo

**Affiliations:** Physical Education and Sports, Faculty of Education, University of Alicante, 03690 Alicante, Spain; lamberto.villalon@ua.es (L.V.-G.); alfonso.penichet@ua.es (A.P.-T.); sergio.sebastia@ua.es (S.S.-A.); j.olmedo@ua.es (J.M.J.-O.)

**Keywords:** back squat, countermovement jump, sports performance, PAP, RM, training

## Abstract

The purpose of this study was to verify if a conditioning activity was effective to elicit postactivation performance enhancement (PAPE) and to increase the performance in vertical jump (VJ) in elite female volleyball players. Eleven national Superliga-2 volleyball players (22.6 ± 3.5 years) were randomly assigned to an experimental and control group. Countermovement jumps (CMJ) were performed on eight occasions: before (Pre-PAPE) and after activation (Post-PAPE), after the match (Pre-Match), and after each of the five-match sets (Set 1 to 5). ANOVA showed significantly increased jump performance for the experiment between baseline (Pre-PAPE) and all the following tests: +1.3 cm (Post-PAPE), +3.0 cm (Pre-Match), +4.8 cm (Set 1), +7.3 cm (Set 2), +5.1 cm (Set 3), +3.6 cm (Set 4), and +4.0 cm (Set 5), all showing medium to large effect size (0.7 < ES < 2.4). The performance of the control group did not show significant increases until Set 3 (+3.2 cm) and Set 5 (+2.9 cm), although jump heights were always lower for the control group than the experimental. The use of conditioning activity generates increased VJ performance in Post-PAPE tests and elicited larger PAPE effects that remain until the second set of a volleyball match.

## 1. Introduction

Vertical jump (VJ) is a good prognosticator of performance in numerous sports that involve explosive actions, including volleyball [1]. The jump height reached by players can be considered a key factor in volleyball. An improvement in height in VJ allows obtaining enhancements in technical actions such as sets, hits, services, or blocks [2] which are decisive to achieve success in a volleyball game [3]. Service, attack, and block effectiveness are the skills more correlated with winning games in volleyball [4,5,6].

In addition, jumping capacity is correlated to muscular strength [7] since greater muscular strength can lead to modifications in force–time profile resulting in better VJ performance. Numerous strength training methods have been used to improve VJ performance in volleyball, being most of them strength-based methods such as plyometrics, combined training methods as contrast and complex training [8], or routines based on weightlifting and powerlifting [9].

While these VJ improvement methods are long-term effect procedures, other practices are aiming at achieving acute effects on performance, on certain occasions during the competition, (e.g., warm-up). One of these short-term methods to enhance VJ performance is the Postactivation Performance Enhancement (PAPE) [10,11,12]. This concept has recently been proposed to be used when high-intensity voluntary conditioning contractions lead to enhancement in voluntary muscular performance, and therefore activation is produced in different ways as with postactivation potentiation (PAP) [10,11]. Although PAP and PAPE are related, they can be considered as a different phenomenon, since the mechanisms that produce PAP are different from those for PAPE. PAP implies an enhancement in the effectiveness of contraction due to a better pairing of actin and myosin, and is generated by electrostimulation. On the other hand, PAPE is related to phenomena such as muscle temperature, the proportion of water in the muscle fibers, and the number of activated motor units among other causes [12]. Therefore, their effects may appear at different times and intensities [13]. The presence of PAP does not have to imply that PAPE is generated [14], even so, PAPE could be evoked by PAP, or occur simultaneously [12], and there have also been cases where PAPE is produced without PAP, which confirms that the mechanisms that generate these phenomena are different [13].

From an ecological point of view, it seems more precise to use PAPE than PAP to refer to the performance improvement in volleyball, since the dependent variables used to verify its existence are directly related to performance, such as force, speed, or jump [12]. Furthermore, its possible effects last longer and are more applicable to real volleyball match conditions, where, due to the game’s rules [15], it is not possible to generate 8-min strength-related pre-activity before the start of the match, but activations before starting the warm-up that could elicit greater PAPE during the match are plausible. On the other hand, the effects of PAP can also increase sports performance, and in volleyball, this could be achieved by including PAP in resistance workouts that allow obtaining improvements in strength through complex training [16,17].

Since the magnitude of PAPE depends on the levels of fatigue and potentiation [18], the magnitude of the activation will depend on this relation, and therefore, the performance will be increased if the effect of the potentiation is larger than fatigue [19]. This relationship is influenced by other individual factors such as individual physiological characteristics of the subject, experience, age, type of muscular fibers’ distribution (i.e., fast-twitch vs. slow-twitch fibers), maximum strength, strength to power ratio, level of training, among others [20].

The design of the activation protocols will greatly affect the result of the enhancement achieved. A resting period between activation and potentiation elicits better performance [21,22]. Similarly, other determining factors of PAPE are the intensity, volume, and protocols of the activation loads and the intensity of jumps or displacements after the potentiation [23].

It has also been suggested that the best increases in VJ are obtained with strength exercises such as the squat, with protocols of 1 to 3 sets of 1 to 5 repetitions and loads greater than 80% of 1RM, obtaining the best results in between 1 to 9 min after activation [24,25]. In the review carried out on vertical jump improvement, Suchomel et al. (2016) arrive at similar conclusions adding the cumulative fatigue of the athlete as individual factors to those already mentioned.

All of these studies have used both trained and untrained subjects as a sample [26]. In this meta-analysis, it was observed that the greatest effects of PAPE occur between 3 and 7 min in trained subjects, obtaining better results than studies for less than 3 min. Also, for studies carried out between 3 and 12 min or more, always for loads greater than 80% of 1RM and in trained subjects, the same authors noted that the longer times included in other meta-analysis are suitable for untrained subjects with smaller loads, where the effect of fatigue is greater.

However, contradictory results were found in the reviewed literature: the improvements in VJ found by Dobbs et al. (2018) were not statistically significant. In addition, some authors did not find effects on jumping performance after a PAP protocol [14,26,27,28,29,30]. Furthermore, the persistence of PAP is significant only for a limited period of time from 28 s to less than 3 min [31], obtaining the performance peak improvement (PAPE) at 6–20 min [25,32].

After reviewing the studies of PAPE protocols applied to volleyball players, it was found that the samples in all the studies are mostly composed of university or college players [22,33,34,35,36], with most of them being male players. The physiological difference between sexes [37] may elicit different responses to PAPE. In general, male players have greater type II fibers cross-sectional area and shorter twitch contraction times, whereas female players show more fatigue resistance [38]. Therefore, the outcomes of PAPE may be different depending on gender [38]. Thus, there is a lack of studies on female athletes, particularly in elite female players [39]. Furthermore, none of the studies in the literature has been conducted in real game conditions with volleyball players.

Therefore, the purpose of this study was to observe the effects of PAPE throughout a match in professional female volleyball players. The initial hypothesis was that squat-based pre-activation can trigger PAPE which is displayed as an improvement of VJ height 8 min after the application of the activation and that PAPE lasts for several minutes in a volleyball match of female national Spanish Superliga 2 players.

## 2. Materials and Methods

### 2.1. Subjects

Twelve Superliga 2 players of University of Alicante volleyball team volunteered to participate in this study (Table 1). Informed consent was obtained from all subjects involved in the study, who read and signed the document before any action in the study was taken. The study was conducted according to the guidelines of the Declaration of Helsinki [40], and approved by the Ethics Committee of University of Alicante (UA-17 November 2018).

The inclusion criteria were to have 4 years of experience minimum in the practice of volleyball in a national competition and to have previous knowledge in both strength training and half-squat exercise. The exclusion criteria were not to participate in all the tests involved in the study or to suffer injury or illness that prevents the performance of the tests. A control group participant suffered an injury during the experimental process, therefore, she was excluded from the experimental procedure and subsequent analysis.

### 2.2. Instruments

For the determination of the force–velocity profile and the vertical jump height, a linear encoder was used (Chronojump-Boscosystem, Barcelona, Spain). To estimate the vertical jump height, a jump mat was used (Chronojump-Boscosystem, Barcelona, Spain), from which to measure the flight time and, thus, estimate the jump height [41]. Both instruments worked at 1000 Hz.

### 2.3. Procedure

**Figure 1:**
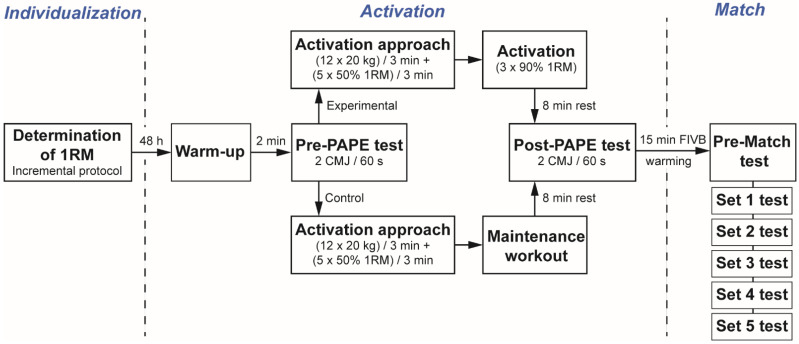
Experimental design of the study¸ RM: Repetition maximum; CMJ: Countermovement jump; PAPE: Post-activation performance enhancement FIVB: Fedération Internationalle de Volleyball.

The experimental design shown in Figure 1 consisted of three phases: individualization, activation, and match, which are described in more detail as follows.

#### 2.3.1. Estimation of 1RM in the Half-Squat Exercise

In order to determine the load corresponding to the 1RM percentage in the half-squat exercise for the PAP protocol, the relationship between force and velocity was analyzed, since the speed of execution and the percentage of 1RM are proportional to each other [41]. An incremental loading test was carried out, in which the initial load was established at 30 kg and was gradually increased in 10 kg steps until mean barbell velocity was below 0.50 m/s (i.e., around 80% of 1RM). Afterward, the load was increased from 5 kg and at the end of the test, with speeds close to 0.30 m/s, increments of 1 kg were made to reach 1RM in the most precise way [42,43]. The value of 1RM was considered the load interpolated in the force–velocity profile with the average acceleration velocity value for the half-squat exercise of 0.30 m/s [44]. The players were refrained from performing physical activity 48 h previous to the test to ensure the absence of fatigue.

#### 2.3.2. Vertical Jump

To determine the possible effect of the activation on PAPE in the lower train, countermovement jump (CMJ) heights were measured using a jump mat [45]. CMJ was performed starting from the standing position, with their feet in the center of the jump mat and hands positioned at the hips in akimbo position. After an auditory signal, subjects performed a knee flexion before jumping vertically to maximum height and were instructed to land in the center of the jump mat. A video camera monitoring players in sagittal plane was used to control that knee flexion reached the right joint angle. Three attempts were carried out with a 60-s resting time [44] and the highest value was considered for data analysis [45].

#### 2.3.3. Activation Protocol

Considering that the sample were players with four years of minimum experience in volleyball training, they can be considered as trained subjects, so the guidelines set by Dobbs et al. (2018) in their meta-analysis were observed, as well as the corresponding 3-repetition activation protocol at 90% intensity of 1RM [35,46] with a resting time of 8 min. Such a protocol follows the margins indicated by these authors [26] and also the rest of the studies consulted [23,24,32,34,45].

Prior to activation, a standardized warm-up was performed for both groups, control and experimental, consisting of 4 min of soft running followed by 4 min of dynamic stretching; then 2 min of speed and changes of rhythm and direction inside the playground, and 5 consecutive CMJ jumps to finish [23]. After the warm-up, 2-min rest period was performed, followed by an initial evaluation of jump height before activation (Pre-PAPE test).

The experimental group performed the activation protocol, consisting of an approaching phase (12 repetitions with 20 kg, 3-min rest, 5 repetitions at 50% of 1RM, 3-min rest), followed by a conditioning phase (3 repetitions at 90% of 1RM). The control group executed the same approaching phase as the experimental group, but when the experimental group performed the conditioning phase control group executed a maintenance workout, consisting of smooth running interspersed with slight changes of direction and 3 vertical jumps. After 8 min, CMJ was measured (Post-PAPE test) to both groups with an identical methodology to that of the Pre-PAP data collection.

#### 2.3.4. PAPE Monitoring during a Volleyball Match

After warm-up was finished, both groups performed a CMJ test before starting the match (Pre-match) and also just at the end of every set of the match (Set 1 to Set 5). This procedure allows describing the evolution in the height reached for volleyball players in a match, as well as to check whether the experimental group shows enhancement derived from PAPE and how much this condition lasts.

### 2.4. Statistical Analysis

Descriptive data are presented as mean and standard deviation. Due to the small sample size, the Shapiro–Wilk normality test was used, which resulted in a normal distribution. The differences in jump height between Pre-PAPE, Post-PAPE, Pre-match, Set 1, Set 2, Set 3, Set 4, and Set 5, in regards to experimental and control groups were evaluated using a repeated-measures ANOVA, including the different tests in time points as an intragroup variable and group as a between-subjects factor. Variance homogeneity and homogeneity of the error variances were verified via the Mauchly’s test (*p* = 0.304) and the Levene’s Test of Equality of Error (*p* range between 0.192 and 0.892 for all comparisons). In addition, a t-test for independent samples was conducted to compare differences on improvement percentage between experimental and control groups. The level of significance was set at *p* ˂ 0.05. The d index was analyzed to determine the magnitude of an effect independent of sample size [47] and to classify the effect size, the criteria of Rhea for elite trained athletes were applied (d < 0.25 trivial; 0.25 ≤ d > 0.50 low; 0.50 ≤ d > 1.0 moderate; d ≥ 1.0 Large) [48]. In this quasi-experimental study, the sample is composed of volleyball elite players competing at a national level. Power analysis conducted with G*Power (v3.1.9.7, Heinrich-Heine-Universität Düsseldorf, Düsseldorf, Germany) indicated a minimum sample size of *n* = 11 subjects in order to detect an effect size of Cohen’s d = 1.6 with 80% power (α = 0.05, two-tailed) [49].

## 3. Results

Table 2 shows the compared results between control and experimental groups for CMJ. There were no significant differences between groups in Pre-PAPE tests in the height reached for both groups (*p*-value > 0.05), indicating that before the intervention the groups were homogeneous. Furthermore, significant intergroup differences and large ES can be observed in CMJ in Post-PAPE, Pre-Match, Set 1, Set 2, and Set 5 always being greater for the experimental group, therefore, the behavior is different for the groups until Set 2 and return to be different in Set 5 but with a reduced ES, Set 3 and Set 4 did not show significant differences. As well, there was a significant difference in the improvement percentage (Δ%) between the control and experimental groups from Post-PAPE until Set 2 test, but in the tests Set 3, Set 4, and Set 5 no differences were found (*p*-value < 0.05).

As it can be observed in Table 3 the ES for the comparison between the pre-intervention and all post-intervention tests always are larger in the experimental group than in control, except in the Pre-PAPE test where both groups have moderate effect sizes, being slightly higher in the control group. However, in the control group, these values are negative, which occurred in a decrease in vertical jump performance.

In Table 3, intragroup differences in CMJ can be appreciated for experimental and control groups. The control group presents lower values for CMJ in the Post-PAPE, Pre-Match, and Sets one to five tests, whereas the experimental group increases the jump height in comparison with Pre-PAPE (baseline) values in all tests. On the other hand, another baseline at the beginning of the match (Pre-Match) allows for the analysis of jump performance in the five sets of the match, which showed different behaviors between both groups. The experimental group showed significant differences with the second set, while the control group did in the third and fifth sets.

**Figure 2:**
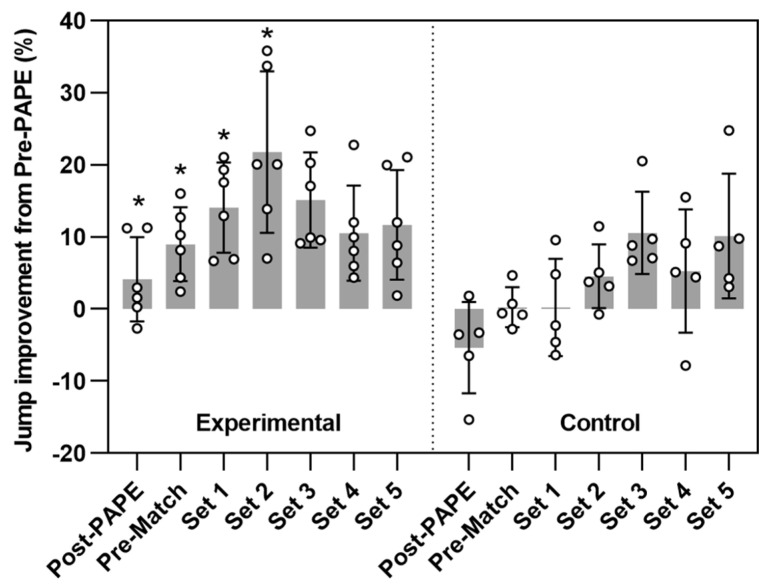
Comparison of the improvement values from Pre-PAPE performance expressed as percentage. * Significant difference between experimental and control groups at the time points: pre-PAPE, Pre-match and Set 1, Set 2, Set 3, Set 4, and Set 5. Bars, whiskers, and dots represent mean, standard deviation and individual values, respectively.

Therefore, the improvement percentage values are lower in the control group than the experimental group as observed in Figure 2. There are significant differences between groups until Set 2. Also, it could be an increase in the values of improvement percentage in the experimental group up to Set 2, where there is a drop in performance until the end of the intervention, Set 5. On the other hand, the control group does not show significant improvement percentages until Set 3.

## 4. Discussion

The aim of this study was to analyze the effects of PAPE for professional female volleyball players during a match. In general, the results highlighted that squat-based pre-activation stimulates higher levels of PAPE shown as improvements in VJ height after activation, these improvements in VJ remained for several minutes during the match. To our knowledge, this is the first study of this kind with elite volleyball players.

PAP is an electrically evoked mechanism that produces an increase of muscle strength and twitch forces, as a result of its contractile history [12,47]. According to this, PAP produces improvements in the rate of force development (RFD) and maximal voluntary contractions (MVC) for a specified level of neural activation [12]. PAP is induced by the rise in the phosphorylation of regulatory light chains, which renders actin-myosin more sensitive to submaximal Ca^2+^ concentrations [48]. This activation occurs with more intensity in the fibers with the isoform II, which are involved in high intensity and short duration actions such as the VJ [19]. Other factors, such as the reduction in the pennation angle after a maximal voluntary contraction, are also suggested as possible mechanisms of PAP.

On the other hand, PAPE is associated whit an intensification in force production induced by previous muscle activity (i.e., voluntary contraction), and its presence is confirmed by performance outcomes [10,12]. Mechanisms proposed for PAPE are different from PAP, nevertheless, there are not well defined yet, but PAPE may be associated whit more lasted processes such as an increase in muscle temperature [49,50]. Also, the Muscle flow or/and water content and muscle activation (Partly through motivation) are mechanisms proposed for PAPE [12]. Finally, the increase in plasma catecholamines induced by exercise [51], and intensification in excitability of high order motor units [48,52,53] are proposed as mechanisms of PAPE, their effects may be observed until 20 min after Pre-activation at least. However, more investigation is needed in order to confirm those effects.

Most studies showed that PAPE protocol increased the performance in VJ in volleyball players [35,52,53,54,55]. Similar results can be found in our study with elite players as the experimental group showed improvements in VJ performance, while the control group has an opposite trend. Nevertheless, these differences between Pre-PAP and Post-PAPE tests are not statistically significant (*p* > 0.05). These results are in concordance with the study by [26,36] in which improvements in VJ were found, although not statistically significant. Due to the difficulty of access to elite athletes, the low sample size in our study may limit the statistical power to show differences between measures taken before and after activation. However, a moderate effect size of the VJ performance was observed, confirming the jump improvement tendency observed.

Volleyball players usually perform a typical explosive strength workout [56]. Those workouts include intensity loads ranging from 40 to 70% of 1RM, which are far from 90% of 1RM, and therefore, the classification as trained subjects [26,57] must be questioned. intensity loads of 90% of 1RM could produce an excess of fatigue in volleyball players and, as a result, the subjects could become non-responders, according to the criteria of [21,58]. Under these circumstances, the load may not fully adjust to the characteristics of the group, and therefore the response obtained is a smaller quantity than expected. Therefore, it is necessary to individualize the PAPE very carefully in order to adjust the activation intensity and volume loads to the individual characteristics of female volleyball players. Previous studies comparing routines based on peak strength and hypertrophy find that explosive-based workouts generate less fatigue [59].

On the other hand, if the improvement percentage values of both groups are compared, as shown in Figure 2, there are significant differences between control and experimental groups in the improvement reached in the post-PAPE tests. Positive improvement percentage values were observed for the experimental group (4.12%) while the control group showed an opposite trend (−5.37%). In addition, these statistically significant results are consistent with their moderate effect sizes, as depicted in Table 3, showing practical significance for the improvement percentage and the jump height reached in the CMJ in the Post-test. Hence, our study suggests that a conditioning activity would generate a positive effect on VJ performance, i.e., PAPE, as a result of an increase in muscle strength obtained 8 min after activation protocol [29,31,47,52,56,59].

The jumps distribution profile during the match was clearly different for control and experimental groups, which suggests that the effect of the activation could be one of the causes of this difference. After the peak of PAPE had occurred in the Post-PAPE test for the experimental group, the CMJ heights still progress, as shown in Table 2, peaking at Set 2 and reaching the end of the match with values similar to those at the start. These results agree with studies in which CMJ is used to evaluate fatigue after using loads and intensities higher than those used in our study (3 sets of 3 repetitions, 90% 1 RM) [59], in the analyzed study a decrease of 6% is observed immediately after the load, but an increase of 2% is observed in the CMJ 24 h after workout. Significant differences and large effect sizes were observed for all test occasions compared to the Pre-PAP test. However, the control group showed a different trend: all VJ heights were lower than the experimental, only Set 3 and Set 5 showed significant differences in regards to the Pre-PAPE test, and peaking at Set 3, later than experimental. This trend can also be analyzed through improvement percentage, shown in Figure 2. The experimental group achieved larger values and, again, showed a peak in Set 2, followed by a decrease in improvement percentage. The control group did not show improvement until Set 3.

The difference between groups could be explained by the presence of PAPE in the experimental group, which effect would extend beyond the time window of 7–12 min [25,26], increasing the jump performance, and also making the athlete more sensitive to future stimulus. Therefore, if PAP is combined with more explosive actions in warm-up routines, a summative effect may occur and therefore a performance improvement. As a result, the effect of PAPE combined with a standard volleyball warm-up, in which numerous jumps are executed [60], may be effective for elicited VJ enhancements.

The effects of PAPE last longer than those of PAP [12], but as in PAP, these effects will depend on the relationship with accumulated fatigue [11]. For the experimental group, the effects of PAPE could be largest than fatigue until Set 2 and consequently, a better improvement in jump performance than in the control group is observed The possible effects of PAPE were evaluated from activation at times ranging from 2 to 20 min maximum in other protocols [30,36]. In our study, CMJ tests were taken in longer time spans: after activation (8 min), at the beginning of the match (23 min), and in Sets 1 to 5 (46, 68, 95, 120, and 123 min, respectively). The experimental group peaked at Set 2 that occurred at 45 min from the beginning of the match and 68 min from activation, while the control group peaked at 90 min after activation. From Set 3 onwards, both groups appear to have similar conditions, and the values for improvement percentage are similar. The possible effect of accumulated fatigue, in addition to the dissipation of activation, causes the behavior of both groups to be more similar, which could be understood as the effect of PAPE is no longer present in the experimental group from Set 3 to the end of the match (i.e., from 90 to 130 min). Performance improvements are probably not mostly due to PAPE, but it is intuited that PAPE helps to generate a summation effect that produces an increase in the performance in VJ of volleyball players.

However, attributing the improvement percentage exclusively to the effect of PAPE generated by an initial conditioning activity and warm-up would not be entirely correct. Numerous physical and cognitive factors can affect the final performance, which is very difficult to control in a real game situation. For these reasons, the individualization of stimuli is very important. Despite this, the two groups in this study were in similar situations and only the group that performed a previous potentiation obtained a better improvement percentage in all sets with a greater effect size magnitude, and therefore, sports performance will be greater in this group.

The main limitation of this study is the sample size due to limited access to elite players in match conditions. The restricted statistical power because of the sample size in this study may have influenced the significance of some of the statistical comparisons conducted. A post hoc power analysis revealed that, for the lowest effect size of interest observed in the present study (*d* = 0.7), the number of players would have been approximately 25 for each group to obtain statistical power at the recommended 80% level. The results of this study serve as a basis that can be generalized to larger populations. Thus, more investigation with larger samples is needed to determine the effects of PAPE in volleyball female players and related sports.

## 5. Conclusions

The use of conditioning activity consisting of three repetitions of 90% of 1RM in the back half-squat exercise generates differences in the increase in CMJ heights between control and experimental group in Post-PAPE tests and elicited larger PAPE effects that remain until the second set of a volleyball match. The results of this study suggest that, if the activation is fitted individually on the correct form, and combined with an optimal warm-up, PAPE may be used to improve vertical jump performance, a key feature in volleyball and other related sports. Therefore, the inclusion of such protocols in volleyball warm-ups should be considered by coaches and physical trainers of volleyball teams. However, further investigation should be carried out, following different warm-up strategies with a wider sample in order to generalize the results achieved in the present study.

## Figures and Tables

**Table 1:** Characteristics of the subjects aggregated by group (mean ± SD).

	Experimental (*n* = 6)	Control (*n* = 5)	Total
Age (years)	21.33 ± 3.0	23.2 ± 3.8	22.2 ± 3.3
Height (cm)	171.3 ± 7.0	172.4 ± 8.7	171.8 ± 7.8
Body mass (kg)	64.0 ± 5.3	63.0 ± 3.8	63.5 ± 4.5
BMI (kg/m^2^)	21.8 ± 5.3	21.3 ± 2.0	21.6 ± 1.6
Volleyball Experience (years)	8.8 ± 2.7	11.0 ± 2.6	9.8 ± 2.7
Strength Experience (years)	3.2 ± 1.8	3.2 ± 2.0	3.2 ± 1.9

BMI: body mass index, *n*: number of subjects, Volleyball experience: years the subjects have been playing volleyball; strength experience: time that subjects have been doing specific workouts.

**Table 2:** Vertical jump height performance (mean ± SD).

	CMJ Experimental (cm) *n* = 6	CMJ Control (cm) *n* = 5	*p*	ES (*d*)
Pre-PAPE	34.08 ± 3.98	31.35 ± 4.28	0.302	0.66 [Moderate]
Post-PAPE	35.40 ± 3.69 *	29.61 ± 4.10	0.036	1.49 [Large]
Pre-Match	37.10 ± 4.09 *#	31.38 ± 3.99	0.045	1.41 [Large]
Set 1	38.84 ± 4.74 *#	31.22 ±2.61	0.011	1.94 [Large]
Set 2	41.37 ± 4.91 *#	32.75 ±4.47	0.015	1.83 [Large]
Set 3	39.15 ± 4.19 #	34.60 ± 4.43 #	0.115	1.05 [Large]
Set 4	37.66 ± 3.98 #	32.76 ± 2.44	0.073	1.23 [Large]
Set 5	38.11 ± 5.40 *#	34.32 ± 3.26 #	0.205	0.83 [Moderate]

* Significant difference between control and experimental groups at the same time point (*p* ˂ 0.05); # Intragroup significant difference between Pre-PAPE and the other post-intervention tests.

**Table 3:** Effect size of intragroup differences in CMJ for Pre-PAPE and Pre-Match vs. the rest of the tests for control and experimental groups.

	Experimental		Control	
	*p*	ES (*d*)	*p*	ES (*d*)
Pre-PAPE vs. Post-PAPE	0.147	0.70 [Moderate]	0.127	0.87 [Moderate]
Pre-PAPE vs. Pre-Match	0.005	1.94 [Large] #	0.922	0.04 [Trivial]
Pre-PAPE vs. Set 1	0.002	2.31 [Large] #	0.903	0.05 [Trivial]
Pre-PAPE vs. Set 2	0.004	2.08 [Large] #	0.069	1.10 [Large]
Pre-PAPE vs. Set 3	0.002	2.40 [Large] #	0.009	2.14 [Large] #
Pre-PAPE vs. Set 4	0.012	1.60 [Large] #	0.313	0.51 [Moderate]
Pre-PAPE vs. Set 5	0.013	1.53 [Large] #	0.046	1.28 [Large] #
Pre-Match vs. Set 1	0.106	0.62 [Moderate]	0.834	0.09 [Trivial]
Pre-Match vs. Set 2	0.022	1.50 [Large] #	0.050	0.74 [Moderate]
Pre-Match vs. Set 3	0.057	0.79 [Moderate]	0.003	1.76 [Large] #
Pre-Match vs. Set 4	0.508	0.01 [Trivial]	0.268	0.76 [Moderate]
Pre-Match vs. Set 5	0.503	0.36 [Low]	0.015	1.61 [Large] #

# Intragroup Significant difference between Pre-PAPE and Pre -Match with the rest of post-intervention tests.

## Data Availability

Data can be obtained through the corresponding author on reasonable request.
